# Influence of resilience on autonomic nervous system habituation to repeated stress exposure: Insights from heart rate variability and heart rate response

**DOI:** 10.1016/j.cpnec.2026.100349

**Published:** 2026-04-25

**Authors:** Christoph Rösner, Husein Maryam, Oliver Tüscher, Katja Petrowski

**Affiliations:** a Medical Psychology & Medical Sociology, University Medical Center of the Johannes Gutenberg - University Mainz, Duesbergweg 6, 55128, Mainz, Germany; b Leibniz Institute for Resilience Research (LIR) gGmbH Mainz, Wallstr. 7, 55122, Mainz, Germany; c Department of Psychiatry and Psychotherapy, University Medical Center of the Johannes Gutenberg - University Mainz, Untere Zahlbacher Str. 8, 55131, Mainz, Germany; d Institute of Molecular Biology gGmbH Mainz, Ackermannweg 4, 55128, Mainz, Germany; e Department of Psychiatry, Psychotherapy and Psychosomatic Medicine University Medicine Halle (Saale), Martin Luther University Halle-Wittenberg (MLU), Halle (Saale), Germany

**Keywords:** Autonomous nervous system (ANS), Heart rate, Heart rate variability, Resilience, Trier social stress test (TSST), Habituation

## Abstract

**Background and objectives:**

This study examines resilience's role in modulating autonomic nervous system (ANS) responses to repeated psychosocial stress, assessed via heart rate variability (HRV) and heart rate (HR) changes.

**Design and methods:**

Sixty healthy males completed the Trier Social Stress Test (TSST) across four sessions, each with six stress phases, to evaluate acute stress response and physiological habituation. Resilience, measured by the Brief Resilience Scale (BRS), was analyzed in relation to HR, HRV indices.

**Results:**

Resilient individuals exhibited better physiological recovery after acute stressor, with increased RMSSD and SDNN post-stress and reduced HR, peak HR, and delta HR for repeated stressor. While HR parameters habituated to repeated stress, anticipatory anxiety (pre-TSST STAI) increased, highlighting a distinction between physiological adaptation and psychological stress anticipation. Despite RMSSD, SDNN and LF recovery after acute stressor, resilience did not significantly impact high-frequency (HF) power.

**Conclusion:**

Resilience appears to enhance physiological recovery after acute stressor and adaptive physiological regulation under repeated stress, supporting its role as a protective factor. These findings have implications for interventions aimed at strengthening stress resilience and reducing allostatic load.

## Highlights

• Resilience improves autonomic recovery during repeated stress, enhancing HRV and HR metrics.
• Resilient individuals show greater parasympathetic engagement during post-stressor recovery.
• Repeated stress exposures reveal resilience-linked habituation in heart rate responses.
• Study findings underscore resilience's protective role in managing repeated psychosocial stress.

## 1 Introduction

Stress is a universal human experience, yet responses to repeated or prolonged stressors do not always follow a uniform course. After the 2010–2011 Christchurch earthquake sequence in New Zealand, community surveys revealed that psychological distress decreased over time, suggesting a habituation of stress responses [1]. However, this decline was not consistent across all communities, as some areas exhibited more persistent distress than others. Such variability underscores the role of resilience and contextual factors in shaping trajectories of adaptation [2,3]. Prolonged exposure to stress is a recognized risk factor for depression, anxiety, and other stress-related disorders [4], which makes it important to examine the mechanisms underlying successful stress adaptation.

Resilience may be instrumental in the absence or the recovery from a temporary occurrence of illness despite significant adversity [3,5]. Resilience, in the psychological sense, is defined as the ability to maintain mental health or recover despite significant adversity [3,[5], [6], [7]]. The resilience concept evolved over time from merely describing whimsical "invincibility" to an opportunity to develop interventions in the clinical setting and the envisioning of resilience research on multiple systems such as the neurobiological system [8]. This is accompanied by measurements of physiological mechanisms related to resilience, as well as consideration of top-down processes such as cognitive appraisal and emotion regulation that can shape stress perception and influence physiological responses [5]. One physiological mechanism that has been explicitly proposed as a pathway linking resilience to health is stress habituation [9]. Bennett and colleagues argue that resilience processes may operate not only through acute stress regulation but also by facilitating adaptive reductions in stress reactivity over repeated exposures. This positions habituation as a critical bridge between short-term coping and long-term resilience outcomes. While acute stress reactivity reflects immediate regulatory capacity, habituation reflects longer-term adaptation and learning. Examining both processes jointly is therefore essential to understand how resilience contributes to sustained health outcomes. Habituation to a repeated stressor occurs recognizably through the physiological systems associated with the stress response, such as the sympathetic and parasympathetic systems, the immune system, and the hypothalamic-pituitary-adrenal axis [10]. This process typically unfolds gradually over repeated exposures and may initially involve substantial physiological costs, such as sustained activation of stress-mediating systems during the early adaptation phase [11]. Once habituation is established, however, stress reactivity diminishes, thereby lowering the physiological demands placed on the individual and freeing resources for more effective coping. This principle of the physiological demands of a stressor is also called Allostatic Load and has the potential to become clinically relevant when physiological resources are exceeded, consequently being termed Allostatic Overload [4,12]. Allostatic Overload poses a risk for several conditions, such as cardiovascular disease or mood and anxiety disorders or greater frailty in the elderly [13]. Because habituation reduces the physiological demands of a stressor, over time habituation leads to a reduced Allostatic Load and thus prevents Allostatic Overload.

The ability of an individual to remain healthy despite adversity as linked to being resilient and stress habituation needs to be tested with several physiological systems as described before. There are multiple physiological systems linked to habituation and Allostatic Load [10]. Research has shown that resilience may influence hypothalamic–pituitary–adrenal (HPA) axis habituation and autonomic nervous system (ANS), measured by skin conductance in the context of repeated stress measurements [14,15]. Furthermore, there is a growing literature directly examining cardiovascular stress reactivity habituation. Hughes, Lu, and Howard [16] provide a conceptual and empirical overview of cardiovascular stress-response adaptation, demonstrating that cardiovascular reactivity often decreases across repeated stress exposures and that these patterns have implications for long-term disease processes. Integrating this framework with resilience research highlights the need to examine not only whether resilient individuals show adaptive habituation but also whether such adaptations confer health protection. For example, highly resilient individuals show a higher reduction in systolic and diastolic blood pressure than individuals with little resilience [17].

Another link might exist between resilience and the stress habituation of the ANS, for example, measured by the cardiovascular system [9,18]. The cardiovascular system is regulated by the ANS via the sympathetic and parasympathetic nervous systems. A healthy handling of stressful situations manifests itself in a phasic Heart Rate Variability (HRV) reduction, which stands for a balanced ANS [19]. Low HRV is represented with mental illnesses such as depression [20] or anxiety disorders [19], among others. In the context of acute stress reactivity, more resilient males show a sympathetic dominance during a stressor and parasympathetic dominance during recovery after a stressor [21]. Furthermore, highly and moderately resilient individuals can be distinguished based on their HRV during cognitively demanding and, thus, more stressful and less demanding tasks [22]. However, these results were obtained with a single stressor, so no conclusions can be drawn about possible habituation. Importantly, most previous studies have focused either on acute stress responses or on habituation processes in isolation. Consequently, the understanding of whether resilience exerts consistent effects across both short-term and repeated stress dynamics remains limited. Furthermore, incorporating the psychological experience of the stressor may provide additional insight into adaptive processes, including learning effects such as anticipation. The present study therefore builds directly on Bennett et al.’s [9] proposal that resilience should be examined in relation to stress habituation, while also extending cardiovascular habituation frameworks [16] into the resilience domain. Cardiovascular markers, in particular, provide a sensitive and temporally precise index of sympathetic and parasympathetic dynamics, making them well suited to capture both immediate stress responses and their adaptation across repeated exposures. To sum up it can be suggested that resilience can modulate both immediate physiological responses to stress (acute reactivity) and longer-term adaptive changes across repeated exposures (habituation), making it important to examine both processes in the present study.

In order to test these relationships, it is necessary to perform a repeated stressor study that captures changes in stress reactivity over time as well as initial acute responses. We therefore hypothesize that (1) higher resilience predicts greater acute sympathetic mobilization during initial stress exposure. This is reflected by larger increases in HR and decreases in HRV, followed by faster parasympathetic recovery, indicated by a more rapid return of HR and HRV toward baseline levels. Additionally, (2) higher resilience predicts greater habituation of cardiovascular stress reactivity across repeated stress exposures, reflected in progressively reduced HR increases and smaller HRV decreases across repeated stress trials.

## 2 Method

### 2.1 Participants

The sample (n = 60) included young, healthy male adults from Germany. A total of 69 participants were initially enrolled in the study. Attrition occurred over the course of the observation period, with three participants discontinuing on test day 1, five on test day 2, and one on test day 3. All subjects received prior information about the procedure and provided written informed consent prior to participation. Because cardiac autonomic function, as reflected in HR and HRV, is influenced by age, cardiovascular and metabolic health, medication use, and lifestyle factors such as smoking and physical fitness [[23], [24], [25]], we recruited only healthy young adults aged 18–30 years to minimize physiological variability unrelated to the experimental manipulations. To avoid confounding influences of sex differences on HRV [26], we restricted our sample to male participants. A brief description of the sociodemographic characteristics of this sample is provided in Table 1. Additional descriptive information on sports activity and sleep variables is provided in the Supplementary Material for interested readers. The participants were recruited via posters and online channels and received a compensation of 75 euros. All subjects completed a preliminary telephone screening for health assessment focusing on the predefined exclusion criteria of this study (such as chronic illness, psychiatric disorders, dependency on alcohol or drugs, smoking ≥ 9 cigarettes daily, body mass index [BMI] not between 18 and 27 kg/m^2^, age range not between 18 and 30, medication [e.g., anti-depressants, beta blockers], familiarity with stress tests). For sample size estimation, effect sizes from previous publications were considered, which indicated small to medium effects [[27], [28], [29]]. Given that HRV studies typically require larger samples, we followed the recommendations by Quintana [30], resulting in a targeted sample size of 61 participants to detect a medium effect size with an α error of 0.05 and 80% power.

**Table 1:** Sample characteristics. Mean values (M), standard deviations (SD) and range for Visual Analogue Scale 1 (“The situation appeared threatening to me”), Visual Analogue Scale 2 (“The situation was stressful for me”), State-Trait Anxiety Inventory (STAI) and Brief Resilience Scale. Participant counts vary across variables (e.g. VAS1) due to occasional missing data caused by problems in data acquisition. Detailed distributions of sports activity and sleep variables are reported in the Supplementary Material.

Variable	n	M	SD	range
Age (in years)	59	24.70	2.89	19-30
BMI (kg/m2)	59	23.40	2.38	18.2-27.8
Doing sports regularly	45			
Sleep problems (e.g. sleep onset and maintenance problems)	11			
In a relationship	35			
**Professional degree**				
Student or apprentice	26			
Professional school diploma	8			
Bachelor/Master/PhD	20			
Without vocational qualification	6			
**Employment**				
Employed	17			
Mini-job	26			
Unemployed	8			
None (e.g. student without Mini-Job)	9			
**VAS1**				
Day 1	58	37.7	31.9	1-100
Day 2	58	29.9	28.3	1-100
Day 3	57	24.8	26.3	1-86
Day 4	58	22.8	25.3	1-94
**VAS2**				
Day 1	58	70.7	24.9	5-100
Day 2	58	52.0	30.9	1-100
Day 3	57	50.0	31.1	1-100
Day 4	58	44.8	27.8	1-100
**STAI-Pre TSST**				
Day 1	58	32.5	5.22	23-45
Day 2	58	36.3	8.01	25-61
Day 3	57	37.6	8.33	25-60
Day 4	58	36.2	7.69	21-59
**STAI-Post TSST**				
Day 1	58	46.7	11.1	28-74
Day 2	58	39.9	9.69	25-66
Day 3	57	38.5	9.37	22-66
Day 4	58	37.4	8.21	21-59
**Brief Resilience Scale (TSST1)**	60	3.69	0.69	1.33-5.00
**Brief Resilience Scale (TSST4)**	60	3.57	0.76	1.33-5.00

### 2.2 Procedure (Stress test)

**Fig. 1:**
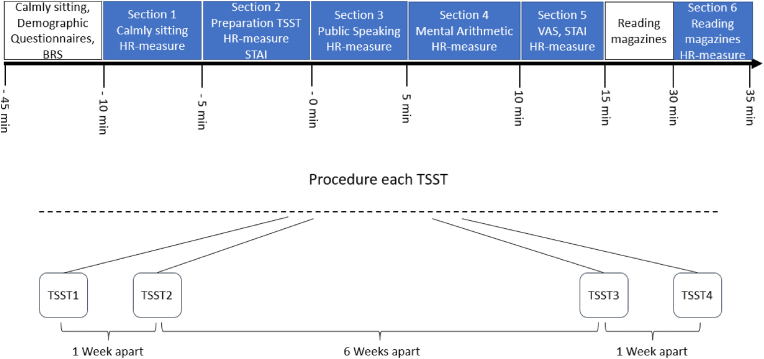
Study design. Blue Boxes indicate Heart Rate measurements. Demographic Questionnaires were filled out once during the first TSST. The BRS was measured at first and last TSST. Note. TSST = Trier Social Stress Test, BRS = Brief Resilience Scale, HR = Heart Rate, STAI = State-Trait Anxiety Inventory, VAS = Visual Analogue Scale.

The participants were scheduled individually using a within-subject experimental study design. The participants were instructed via e-mail to refrain from alcohol consumption and any strenuous physical activity/exercise 24 h prior to laboratory days and from eating or drinking 1 h before their laboratory visits. For an overview of the study design, see Fig. 1. Upon arrival, participants sat down for 45-min as an adaption phase, during which they completed demographic questionnaires and the Brief Resilience Scale (Smith et al., 2008, description see below), before they were introduced to the Trier Social Stress Test (TSST) by Kirschbaum, Pirke, & Hellhammer [31]. The TSST is a well-established social evaluative stressor, as it reliably elicits robust and ecologically valid psychophysiological stress responses. In addition, the TSST captures key features of everyday stress experiences such as social evaluation, uncontrollability, and performance pressure, thereby enhancing the generalizability of findings to real life contexts. The psychosocial stress protocol includes preparation time (5 min) followed by a 5-min job interview situation and a subsequent 5-min mental arithmetic task in front of a two-person-panel [32,33]. Testing took on average 2 h to complete on each day of laboratory assessment. Participants were seated during the baseline and preparation phases of the Trier Social Stress Test, stood while performing the interview and mental arithmetic tasks, and were seated again during the post-stress recovery period. For the measurement of a repeated stressor the TSST was conducted four times (t1-t4) for every participant. The second TSST measurement day occurred 1 week after the first, the third measurement day after 7 weeks, and the fourth measurement day after 8 weeks. It is important to note that habituation to psychosocial stress is influenced by the spacing of stress exposures. Cortisol responses seem to habituate across repeated TSST administrations, but that the degree of habituation depends on the time interval between sessions. Shorter intervals are associated with stronger habituation, whereas longer intervals allow for partial recovery of the stress response [34]. By incorporating a six-week gap, our study design enables examination of whether resilience-related habituation effects extend beyond short-term adaptation and are preserved over a longer temporal interval. To avoid possible effects of the participants’ presenting a speech by heart, getting used to the panel or memorizing the arithmetic task, the setting of the stress test was minimally altered [34]. The job description was modified each time, at least one panel member was exchanged, and the arithmetic task with the initial numbers were changed using a different starting number and subtrahend. For each TSST, participants completed the Visual Analogue Scale and the State-Trait Anxiety Inventory (Laux et al., 1981; descriptions see below) to assess stress induction based on subjective self-assessment. The questionnaires were filled out electronically using SoSci Survey version 3.2.06. In addition to the autonomic and subjective measures reported here, salivary cortisol was collected as part of the broader study protocol in a separate subsample, whereas salivary alpha-amylase (sAA) was not assessed; the corresponding cortisol findings are reported separately [15].

### 2.3 Heart rate and heart rate variability

For data acquisition, the wireless mobile system EQuiVital SEM Module with chest harness was used to record a 2-channel ECG, respiration rate and acceleration with 256 Hz. Simultaneously, live monitoring was carried out and recorded with time markers using the software LabChart 8 (ADInstruments Pty Ltd.). ECG was assessed using disposable electrodes (Dahlhausen & Co. GmbH, Cologne, ECG electrodes type 460). For data acquisition and processing we followed expert guidelines [35] and defined standards by the Task Force of The European Society of Cardiology and the North American Society for Pacing and Electrophysiology [36]. For a detailed analysis of the HRV data, the TSST measurements were divided into six sections each lasting 5 min (Fig. 1). The division into 5-min intervals for investigating HRV data has also been adopted by other researchers [19,37]. The first 5-min section (section 1 = Resting Period) was the baseline resting phase of calmly sitting down, conducted within the adaption phase after the first 35 min (see procedure).

A comfortable sitting position was chosen for each participant. In this phase the participants were instructed to breathe normally without talking and moving around. The second section (section 2 = Preparation TSST) was for the participant to prepare the TSST speaking task. The third and fourth section (section 3 = Public Speaking; section 4 = Mental Arithmetic) were the speaking task and the arithmetic task, both performed by the participant in front of a board of two persons. The last two sections (section 5 = Post TSST 1; section 6 = Post TSST 2) were both post TSST resting phases with the first immediately after the TSST and the second 30 min after the TSST. The raw files (HR) were corrected via the Polar LabChart 8 and the add-on HRV module (ADInstruments Pty Ltd.). The automatic beat detection was used to find the QRS complex and the corresponding R-R intervals between heart beats. This was followed by manual visual inspection of the three phases as the gold standard compared to automatic artefact corrections. Importantly, HRV parameter are sensitive towards movements, respiration (respiratory sinus arrhythmia), and artifacts of physiological or technical origins [35]. Ectopic beats were excluded and artifacts manually checked for correct beat-to-beat R-R-interval detection (for instance erroneous values of the R-R-interval) in the post-processing of the recordings. A total of 1181 beats were manually corrected. Trained medical staff extracted values from these recording phases and saved them to import data into SPSS Statistics (IBM, Chicago, IL, USA). For heart rate analysis, we also included additional variables: peak heart rate, baseline-to-peak heart rate (section 1 to Peak), and peak-to-end heart rate (Peak to section 6), alongside overall heart rate measures. For the HRV analyses time and frequency domain variables were collected. The time domain variable RMSSD (square root of the mean of the squares of the differences of subsequent NN intervals) represents short-term fluctuations in milliseconds (ms) of parasympathetic nervous system activity. SDNN (standard deviation of the NN intervall) is also a time-domain variable and reflects all the cyclic components responsible for variability in the period of recording. RMSSD and SDNN are therefore important HRV-variables. For the spectral analysis of certain frequency bands, Low Frequency (LF), High Frequency (HF) and the ratio (LF/HF) was used. HF power (0.15–0.40 Hz) is widely interpreted as an index of parasympathetic (vagal) activity, while LF power (0.04–0.15 Hz) reflects a mixture of sympathetic and parasympathetic influences, including baroreflex modulation [38]. The LF/HF ratio has traditionally been treated as a marker of sympathovagal balance; however, recent work highlights its limitations—LF/HF interpretations can be confounded by respiration, heart rate, and the nonlinear nature of autonomic interactions, and may be misleading if taken as a simple gauge of sympathetic–parasympathetic balance [39,40].

### 2.4 Questionnaires

Resilience was measured by using the German version [41,42] of the Brief Resilience Scale (BRS; Smith et al., 2008) at baseline and at the last measurement day. Compared to other questionnaires and distinctly no trait like resilience factors, the BSR assesses resilience more closely as an outcome as currently defined in the field [3,5,43], namely the individual ability to recover from stress despite significant adversity (e.g., chronic stressors or adverse life events). The six items of the BRS are rated on a five-point Likert scale (1 = strongly disagree, 2 = disagree, 3 = neutral, 4 = agree, 5 = strongly agree), e.g. “I usually come through difficult times with little trouble”. Translated into German in 2018, the BRS had shown a good reliability (α = .85) in two different samples (N1 = 1128, N2 = 1481, Chmitorz et al., 2018) and also good reliability (α = .87, ω = 0.87) in a newer validation (N = 2522, Rösner, Brähler et al., 2024). In factor analysis, the BRS resulted in a single-factor solution. BRS has a low to moderate relationship with optimism and internal locus of control and correlates high with self-efficacy but represents an independent construct after confirmatory factor analysis, as one factor emerges for each of these constructs [41,44].

The Visual Analogue Scale (VAS) was employed as a subjective evaluation tool following each TSST on all four days. Scores range from 0 to 100, with higher values indicating stronger agreement. The VAS items included: VAS1, “The situation appeared threatening to me”; VAS2, “The situation was stressful for me”. The VAS was completed after the TSST on each of the four measurement days.

The State-Trait Anxiety Inventory (STAI, [45]) was used to assess participants' anxiety levels, specifically focusing on the state subscale. The STAI is a validated self-report questionnaire that includes two subscales measuring trait and state anxiety; however, only the State Anxiety Scale (S-Anxiety) was used in this study. This 20-item subscale evaluates participants' anxiety levels "right now, at this moment," allowing for assessment of situational, transient anxiety in response to experimental tasks. Participants rated their responses on a 4-point Likert scale, with higher scores indicating greater levels of current anxiety. This measure was chosen to capture momentary anxiety responses directly related to the study conditions. Participants completed the STAI immediately before and after the TSST on each of the four measurement days.

### 2.5 Statistical analysis

To assess stress induction based on self-assessment, mean values and standard deviations were calculated and presented for the VAS. Similarly, mean values and standard deviations were reported for the STAI. Repeated measures ANOVA was conducted on STAI scores before and after each TSST to evaluate changes in anxiety levels for each separate TSST measurement day. Furthermore, repeated measures ANOVA were conducted for differences between TSST measurement days regarding STAI before TSST and after TSST, as well as VAS.

To determine whether the selected stressor had any effect, a repeated-measures ANOVA was conducted comparing the average heart rate (beats per minute, BPM) between section 1 (Resting Phase) and section 3 (Public Speaking). The main statistical analysis was performed using mixed models. First, it was checked whether the influence of resilience (measured at baseline) on HRV parameters differed among the six sections. Then, it was checked whether the influence of resilience (measured at baseline) on HRV parameters differed at the different measurement days. The interaction of time and resilience was set as a fixed effect for both calculations, whereby intra-individually intercept was set as random effect. This allowed to check whether the effects of resilience on the HRV parameters differed at the four measurement days and at the six sections. Furthermore, *R*^*2*^ is reported as the goodness-of-fit measure of the models. All analysis were conducted using jamovi [46].

## 3 Results

The average BRS score in our sample was M = 3.69 (SD = 0.69, min = 1.33, max = 5.00), which is comparable to the German normative data (e.g., M = 3.54 at the 50th percentile across the general population, and M = 3.70 for males aged 25–34; Rösner et al., 2024). This suggests that the participants in our study exhibited average levels of psychological resilience. For the second BRS measurement on the final measurement day, the mean was 3.57 (SD = 0.76). A paired-samples *t*-test comparing the first and second BRS measurements revealed no significant difference, *t*(57) = 1.55, p = .127. These results indicate that participants’ resilience scores remained stable across the study period.

### 3.1 Physiological and psychological reactions to the stressor

**Fig. 2:**
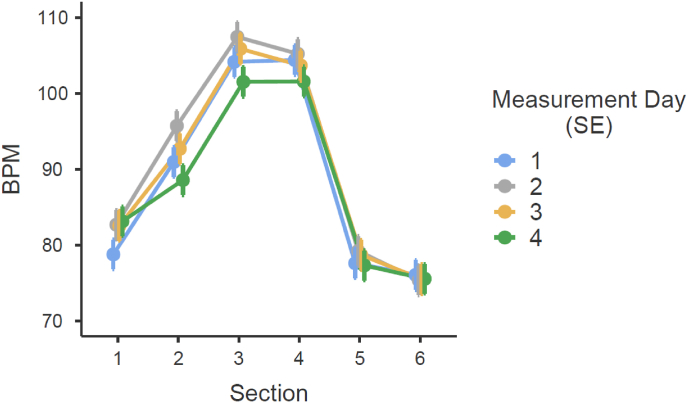
Average heartbeats per minute (BPM) during each TSST section for each TSST measurement day (Measurement 1-4) with standard errors (SE). Description of the sections: section 1 = baseline (35-40 min of 45 min adaption period); section 2 = preparation/anticipation; section 3 = speech task; section 4 = arithmetic task; section 5 = post task (0-5 min after completion of TSST); section 6 = 30 min post task (30-35 min after competition TSST).

**Fig. 3:**
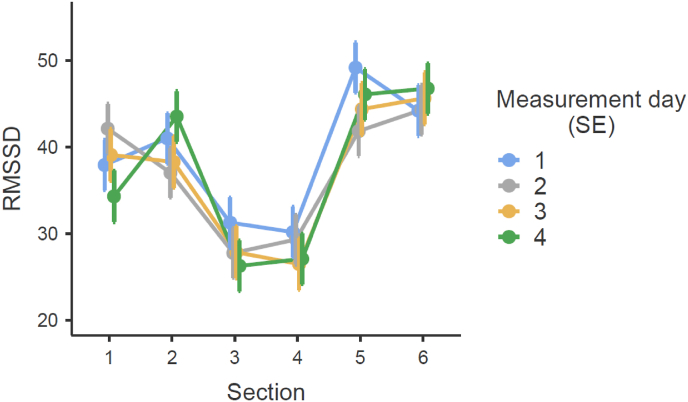
Progression of RMSSD across the six TSST sections for each of the four TSST measurement days with standard errors (SE). Description of the sections: section 1 = baseline (35-40 min of 45 min adaption period); section 2 = preparation/anticipation; section 3 = speech task; section 4 = arithmetic task; section 5 = post task (0-5 min after completion of TSST); section 6 = 30 min post task (30-35 min after competition TSST).

A repeated measures ANOVA was conducted to examine the effects of section and measurement day on heart rate (BPM). The analysis revealed a significant main effect of section on BPM, *F*(5, 1095) = 732.63, *p* < .001, indicating a substantial difference in heart rate across the six sections. Additionally, there was a significant interaction between section and measurement day, *F*(15, 1095) = 2.88, *p* < .001, suggesting that heart rate changes over time varied depending on the section. Post-hoc test revealed significant differences with an increase from section 1 to section 3 for all measurement days: mean difference_day 1_ = 25.55 (p < .001), mean difference_day 2_ = 24.76 (p < .001), mean difference_day 3_ = 23.32 (p < .001) and mean difference_day 4_ = 18.27 (p < .001). Consequently, the TSST effectively increased heart rate. Fig. 2 illustrates that the psychosocial stressor impacted BPM progression for all four TSST measurement days, with an increase observed from section 1 (Pre-TSST) to section 3 (Public Speaking task, TSST). Fig. 3 depicts the progression of RMSSD across the six TSST sections for each of the four experimental sessions, illustrating both within-session dynamics and between-session changes. RMSSD shows a marked decrease during the stressor phases (sections 3, 4), followed by a recovery in the post-stressor phases (sections 5–6).

Table 1 shows mean values and standard deviation for VAS und STAI. The repeated measures ANOVA revealed differences in state anxiety before and after the first TSST F(1, 57) = 15.0, p < .001 and the second TSST F(1, 57) = 44.2, p < .001, but not at the third TSST F(1, 56) = 0.6, p = .461 and fourth TSST F(1, 57) = 1.4, p = .241. Repeated measurement ANOVAs of the Visual Analogue Scale (VAS1) and the State-Trait Anxiety Inventory (STAI) across the four TSST measurement days were conducted (for detailed results of the post-hoc pairwise comparisons between measurement days following repeated-measures ANOVA see Table 2). The omnibus tests conducted through repeated measures ANOVAs revealed significant effects for all measured variables across the four TSST measurement days. For VAS1, the analysis yielded significant results: *F*(3, 168) = 11.8, *p* < .001. VAS2 also showed significant differences, *F*(3, 168) = 24.4, *p* < .001. For the STAI pre-TSST measurements, significant differences were observed as well, *F*(3, 168) = 12.2, *p* < .001. Finally, the STAI post-TSST assessments indicated significant effects, *F*(3, 168) = 36.8, *p* < .001. For VAS1 (“The situation appeared threatening to me”), the first analysis indicated a significant decrease in scores from Day 1 to Day 2, Day 3, and Day 4, with mean differences of −8.42, −12.47, and −15.11, respectively (all *p* < .001). Analysis of VAS2 further confirmed substantial decreases, showing mean differences of −18.60, −19.96, and −25.16 when comparing Day 1 to Days 2, 3, and 4 (all *p* < .001). Conversely, STAI scores showed a significant increase in state anxiety from Day 1 to subsequent days, with mean differences of 3.75, 5.05, and 3.79 when comparing Day 1 to Days 2, 3, and 4 (all *p* < .001) for the pre-TSST measurements, indicating that anxiety levels were higher on these days compared to Day 1. In the post-TSST assessments, significant decreases in state anxiety were observed from Day 1 to Days 2, 3, and 4, with mean differences of −7.05, −8.05, and −9.42, respectively (all *p* < .001), reflecting diminished anxiety levels following the tasks. Notably, there were no significant differences in anxiety levels between Day 2 and Day 3, as well as between Days 3 and 4, suggesting that anxiety responses may stabilize over time.

**Table 2:** Post-hoc pairwise comparisons between measurement days following repeated-measures ANOVA for Visual Analogue Scale 1 (“The situation appeared threatening to me”), Visual Analogue Scale 2 (“The situation was stressful for me”), State-Trait Anxiety Inventory (STAI).

Measure	Comparison	Mean Difference	SE	df	t	p
VAS1	Day 1 vs. Day 2	−8.42	2.43	56.0	3.47	**0.001**
	Day 1 vs. Day 3	−12.47	3.25	56.0	3.83	**< 0.001**
	Day 1 vs. Day 4	−15.11	3.18	56.0	4.76	**< 0.001**
	Day 2 vs. Day 3	−4.05	2.70	56.0	1.50	0.139
	Day 2 vs. Day 4	−6.68	2.45	56.0	2.73	**0.008**
	Day 3 vs. Day 4	−2.63	2.09	56.0	1.26	0.213
VAS2	Day 1 vs. Day 2	−18.60	3.23	56.0	5.75	**< 0.001**
	Day 1 vs. Day 3	−19.96	3.56	56.0	5.61	**< 0.001**
	Day 1 vs. Day 4	−25.16	3.19	56.0	7.88	**< 0.001**
	Day 2 vs. Day 3	−1.37	3.02	56.0	0.45	0.652
	Day 2 vs. Day 4	−6.56	3.45	56.0	1.90	0.063
	Day 3 vs. Day 4	−5.19	2.23	56.0	2.33	**0.024**
STAI Pre-TSST	Day 1 vs. Day 2	3.75	0.993	56.0	−3.78	**< 0.001**
	Day 1 vs. Day 3	5.05	1.080	56.0	−4.68	**< 0.001**
	Day 1 vs. Day 4	3.79	1.002	56.0	−3.78	**< 0.001**
	Day 2 vs. Day 3	1.30	0.782	56.0	−1.66	0.102
	Day 2 vs. Day 4	0.04	0.619	56.0	−0.06	0.955
	Day 3 vs. Day 4	−1.26	0.738	56.0	1.71	0.092
STAI Post-TSST	Day 1 vs. Day 2	−7.05	1.027	56.0	6.87	**< 0.001**
	Day 1 vs. Day 3	−8.05	1.123	56.0	7.17	**< 0.001**
	Day 1 vs. Day 4	−9.42	1.233	56.0	7.64	**< 0.001**
	Day 2 vs. Day 3	−1.00	0.848	56.0	1.18	0.244
	Day 2 vs. Day 4	−2.37	0.842	56.0	2.81	**0.007**
	Day 3 vs. Day 4	−1.37	0.697	56.0	1.96	0.055

#### 3.1.1 Within-TSST sections effects of resilience

**Fig. 4:**
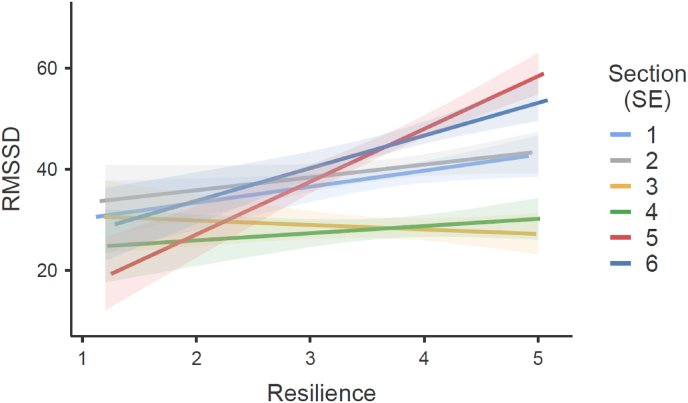
Effects plot from the mixed-effects model showing the association between resilience (BRS) and RMSSD across the six sections of the TSST. The y-axis represents model-predicted RMSSD values, and the plotted lines illustrate the model-estimated relationship between BRS and RMSSD, stratified by TSST section. Error bars indicate standard errors (SE). Section descriptions: section 1 = baseline (35–40 min of the 45-min adaptation period); section 2 = preparation/anticipation; section 3 = speech task; section 4 = arithmetic task; section 5 = post task (0–5 min after completion of the TSST); section 6 = 30 min post task (30–35 min after completion of the TSST).

Mixed models were calculated to examine the influence of resilience (BRS) on Heart Rate, RMSSD, SDNN, LF, HF, and LF/HF across all six TSST sections. The omnibus tests revealed significant effects for Heart Rate, *F*(5, 1284) = 3.24, *p* = .007, *R*^*2*^ = 0.382; RMSSD, *F*(5, 1283) = 4.54, *p* < .001, *R*^*2*^ = 0.113; SDNN, *F*(5, 1284) = 2.44, *p* = .033, *R*^*2*^ = 0.106; and LF, *F*(5, 1284) = 4.66, *p* < .001, *R*^*2*^ = 0.078. No significant effects were found for HF, *F*(5, 1284.9) = 1.26, *p* = .277, or LF/HF, *F*(5, 1284) = 0.88, *p* = .496. Detailed information about parameters with significant results can be found in Table 3. Resilience was associated with increased RMSSD during the immediate post-stressor phase (section 5) compared to baseline (section 1; *β* = 7.26, *p* = .007), indicating higher parasympathetic activity in resilient individuals during recovery (Fig. 4). Similarly, SDNN showed significant increases in section 5 relative to baseline (*β* = 6.33, *p* = .020), suggesting enhanced autonomic flexibility post-stressor for more resilient individuals. Heart rate was also influenced by resilience, with a significant increase during the arithmetic task (section 4) compared to baseline (*β* = 3.98, *p* = .002). Higher resilience was associated with a greater heart rate increase during this task. Low Frequency (LF) power demonstrated resilience effects, showing a significant increase in section 6 compared to baseline (section 1; *β* = 730.05, *p* < .001). This increase in LF suggests a stronger sympathetic response or mixed autonomic engagement in the later post-TSST recovery phase among resilient participants.

**Table 3:** Mixed Model results of the fixed effect parameter of interaction between resilience (BRS) and the measurement days 2 to 1 (2-1), 3 to 1 (3-1), and 4 to 1 (4-1) and Mixed Model results of the fixed effect parameter of interaction between resilience (BRS) and the measurement sections 2 to 1 (2-1), 3 to 1 (3-1), 4 to 1 (4-1), 5 to 1 (5-1) and 6 to 1 (6-1) for Heart Rate, Peak, Delta Baseline to Peak, Delta Peak to End and the HRV-parameters RMSSD, SDNN and LF.

Interaction	β	df	t	p
Section				
RMSSD				
2 - 1 ✻ Resilience	−0.630	1283	−0.236	0.813
3 - 1 ✻ Resilience	−4.056	1283	−1.521	0.129
4 - 1 ✻ Resilience	−1.749	1283	−0.656	0.512
5 - 1 ✻ Resilience	7.258	1283	2.722	**0.007**
6 - 1 ✻ Resilience	3.287	1283	1.232	0.218
SDNN				
2 - 1 ✻ Resilience	1.867	1284	0.685	0.493
3 - 1 ✻ Resilience	−1.576	1284	−0.579	0.563
4 - 1 ✻ Resilience	0.658	1284	0.242	0.809
5 - 1 ✻ Resilience	6.333	1284	2.325	**0.020**
6 - 1 ✻ Resilience	4.786	1284	1.756	0.079
Heart Rate				
2 - 1 ✻ Resilience	0.588	1278	0.463	0.643
3 - 1 ✻ Resilience	2.308	1278	1.818	0.069
4 - 1 ✻ Resilience	3.981	1278	3.136	**0.002**
5 - 1 ✻ Resilience	0.163	1278	0.129	0.898
6 - 1 ✻ Resilience	−0.004	1278	−0.003	0.998
LF				
2 - 1 ✻ Resilience	113.66	1278	0.560	0.575
3 - 1 ✻ Resilience	−97.16	1278	−0.479	0.632
4 - 1 ✻ Resilience	8.39	1278	0.041	0.967
5 - 1 ✻ Resilience	358.32	1278	1.767	0.078
6 - 1 ✻ Resilience	730.05	1278	3.597	**<0.001**
RMSSD				
2 - 1 ✻ Resilience	−0.630	1283	−0.236	0.813
3 - 1 ✻ Resilience	−4.056	1283	−1.521	0.129
4 - 1 ✻ Resilience	−1.749	1283	−0.656	0.512
5 - 1 ✻ Resilience	7.258	1283	2.722	**0.007**
6 - 1 ✻ Resilience	3.287	1283	1.232	0.218
SDNN				
2 - 1 ✻ Resilience	1.867	1284	0.685	0.493
3 - 1 ✻ Resilience	−1.576	1284	−0.579	0.563
4 - 1 ✻ Resilience	0.658	1284	0.242	0.809
5 - 1 ✻ Resilience	6.333	1284	2.325	**0.020**
6 - 1 ✻ Resilience	4.786	1284	1.756	0.079
Measurement day				
Heart Rate				
2 - 1 ✻ Resilience	−0.634	1283	−0.655	0.513
3 - 1 ✻ Resilience	−3.279	1293	−2.831	**0.005**
4 - 1 ✻ Resilience	−0.928	1289	−0.848	0.396
Peak				
2 - 1 ✻ Resilience	−1.851	1293	−2.243	**0.025**
3 - 1 ✻ Resilience	−5.372	1297	−5.429	**<0.001**
4 - 1 ✻ Resilience	−2.539	1294	−2.726	**0.006**
Delta Baseline to Peak				
2 - 1 ✻ Resilience	−0.409	1277	−0.584	0.559
3 - 1 ✻ Resilience	−2.469	1284	−2.940	**0.003**
4 - 1 ✻ Resilience	−1.509	1280	−1.877	0.061
Delta Peak to End				

2 - 1 ✻ Resilience	−1.33	1282	−2.169	**0.030**

3 - 1 ✻ Resilience	−3.33	1287	−4.530	**<0.001**

4 - 1 ✻ Resilience	−2.63	1284	−3.803	**<0.001**

#### 3.1.2 Across-session habituation effects of resilience

**Fig. 5:**
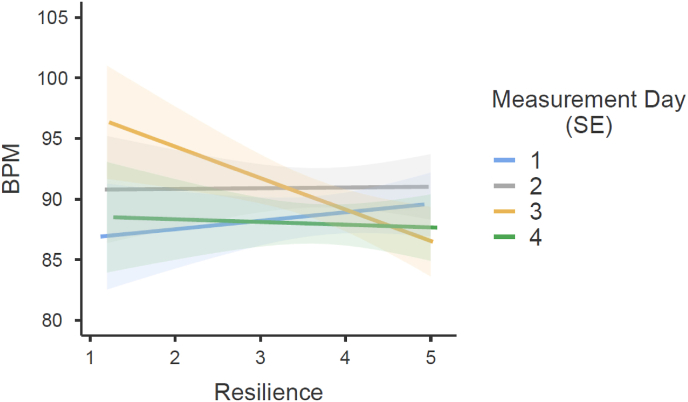
Effects plot from the mixed-effects model showing the association between resilience (BRS) and heart rate (BPM) across all four measurement days. The y-axis displays model-predicted heart rate values, and the plotted lines represent the model-estimated relationship between BRS and heart rate, stratified by measurement day. Error bars indicate standard errors (SE).

Mixed models were calculated to assess the influence of resilience on Heart Rate, Peak, Delta Baseline to Peak, Delta Peak to End, RMSSD, SDNN, LF, HF, and LF/HF across all four TSST measurement days. The omnibus tests revealed significant effects for Heart Rate, *F*(3, 1299) = 2.78, *p* = .040, *R*^*2*^ = 0.005; Peak, *F*(3, 1293) = 9.87, *p* < .001, *R*^*2*^ = 0.017; Delta Baseline to Peak, *F*(3, 1283) = 3.40, *p* = .017, *R*^*2*^ = 0.049; and Delta Peak to End, *F*(3, 1288) = 8.14, *p* < .001, *R*^*2*^ = 0.036. However, there were no significant effects for RMSSD, *F*(3, 1312) = 0.54, *p* = .654; SDNN, *F*(3, 1306) = 1.75, *p* = .154; LF, *F*(3, 1313) = 0.37, *p* = .774; HF, *F*(3, 1322.3) = 0.42, *p* = .738; or LF/HF, *F*(3, 1311) = 1.10, *p* = .349. Detailed information about parameters with significant results can be found in Table 3. Regarding measurement days, resilience was linked to a decrease in heart rate on Day 3 compared to Day 1 (*β* = −3.28, *p* = .005), pointing to a potential habituation effect over repeated exposures (Fig. 5). Additionally, resilient individuals displayed a reduced peak heart rate across Days 2, 3, and 4 relative to Day 1, with the most substantial effect on Day 3 (*β* = −5.37, *p* < .001), indicating an adaptation to stress. In terms of Delta Baseline to Peak changes, resilience was associated with a smaller increase in heart rate from baseline to peak on Day 3 compared to Day 1 (*β* = −2.47, *p* = .003), suggesting that resilient individuals might experience less intense physiological activation during peak stress over repeated sessions. Finally, Delta Peak to End differences showed resilience-related reductions across Days 2, 3, and 4 (all *p* < .05), supporting the idea that resilience buffers the stress response and enhances recovery across repeated stress exposures. These findings imply that resilience contributes to more adaptive physiological regulation during stress and improved habituation over time.

## 4 Discussion

Habituation take place on many different physiological systems [9], wherefore the influence of resilience on habituation can be tested on different physiological systems. The influence of resilience on HRV as a proxy for ANS has already been verified using a single stressor [21,22], but the influence of resilience on Heart Rate and HRV parameters using a repeating stressor in the sense of habituation has not yet been verified, so the influence of resilience on (1) acute stress reactivity and (2) habituation was investigated in the present study. Overall, the results of the present study indicated that resilience was associated with more adaptive regulation, reflected in faster parasympathetic recovery, moderated heart rate responses during acute stress, and enhanced habituation across repeated TSST sessions, suggesting that resilient individuals show both stronger recovery and attenuated stress reactivity over time.

The sample's BRS scores aligned with German norms, indicating average resilience. Repeated measures ANOVAs revealed habituation effects in perceived threat and post-task anxiety, as VAS and post-TSST STAI scores decreased significantly across TSST days, showing reduced threat perception and anxiety levels after each session. Conversely, pre-TSST STAI scores rose over the measurement days, suggesting increased anticipatory anxiety before each stressor, as participants became increasingly aware of the upcoming task demands after their initial exposure. While this could be interpreted as sensitization, the concurrent decline in post-TSST anxiety suggests that experiential stress responses habituated, whereas anticipatory responses followed a distinct trajectory.

Heart rate and HRV metrics further emphasized resilience effects, particularly in acute stress recovery. Resilience was associated with increased RMSSD and SDNN during post-stressor recovery (section 5) with RMSSD indicating enhanced parasympathetic (vagal) activity, while SDNN represents overall heart rate variability encompassing both sympathetic and parasympathetic influences. However, no significant effects were found for HF power, a direct marker of parasympathetic activity, indicating that resilience may support acute stress recovery through other mechanisms beyond HF-linked parasympathetic activation. These results were consistent with previous research showing enhanced parasympathetic recovery in resilient individuals [21], but unlike that study, our data do not provide direct evidence for sympathetic dominance during the stressor. A possible reason for the better parasympathetic activity of more resilient individuals could be the vagus nerve activity, which connects the brain to the heart and is the main nerve of the parasympathetic system [35]. Vagally-mediated HRV indicates increased activity of the vagus nerve and thus indicates increased parasympathetic activity in more resilient individuals [22]. Resilience may enhance vagal activity through adaptive emotion regulation and neural mechanisms that promote physiological flexibility. Resilient individuals often employ adaptive emotion regulation strategies, such as reappraisal, which activate the prefrontal cortex [5]. This activation can enhance vagal tone by facilitating top-down control over the autonomic nervous system, promoting parasympathetic recovery following stress [47]. Additionally, polyvagal theory posits that the ventral vagal complex, associated with social engagement and self-regulation, supports adaptive responses to stress [48]. Therefore, resilience may foster a physiological state conducive to efficient stress recovery, characterized by enhanced vagal activity.

Across measurement days, resilience was associated with lower Heart Rate, peak heart rates and reduced Delta Baseline to Peak changes by Day 3, supporting resilience's role in stress adaptation through reduced physiological activation at peak stress. Additionally, resilient individuals displayed quicker recovery, as indicated by reduced Delta Peak to End changes across days. While quicker recovery would typically be attributed to parasympathetic activation, the absence of significant effects in HF power suggests resilience individuals may have better cardiac autonomic modulation. These findings imply that resilience enhances autonomic regulation during stress, facilitating habituation and improving recovery, consistent with its hypothesized protective effects on stress resilience and health. Interestingly, habituation did not occur uniformly across systems. Subjective measures, including perceived threat (VAS) and post-TSST state anxiety, decreased across sessions, reflecting typical habituation. In contrast, pre-TSST anxiety increased over repeated exposures, suggesting anticipatory sensitization. Autonomic indices, such as HRV, showed only partial or inconsistent across-day changes, indicating that cardiovascular adaptation may follow a different time course than subjective stress ratings. These findings align with previous research demonstrating differential habituation across stress systems. For example, Schommer et al. [49] and Wüst et al. [50] reported that endocrine, autonomic, and subjective stress measures can exhibit distinct adaptation trajectories across repeated stress exposures. Importantly according to the present study results, resilience seems to modulate certain aspects of autonomic adaptation, such as faster post-stressor parasympathetic recovery and attenuated peak heart rate, suggesting that individual differences in resilience can influence the extent and pattern of habituation, even when system-specific adaptation is mixed. The association between resilience and healthy autonomic regulation may be explained by the more effective use of emotion regulation strategies among resilient individuals [51]. The impact of reappraisal, a common emotion regulation strategy, on the autonomic nervous system has been explored in multiple studies, though findings regarding its influence on habituation have been mixed [[52], [53], [54]]. In contrast, suppression, another emotion regulation strategy, appears to negatively affect autonomic stress reactivity and shows a negative correlation with resilience [52].

Applied to the allostatic load model, the relatively diminished activation of indicators of the parasympathetic nervous system in less resilient individuals could indicate a prolonged allostatic response after stressor or allostatic load type 3 [55]. Since less resilient individuals have been found to maintain this stress response with repeated occurrences of the stressor, the stress response could promote Allostatic Overload through prolonged need for physiological resources to cope with the stressor. However, since there is no inter-individually reference frame regarding the necessary length of a stress response to classify it as a prolonged response, this may still be speculation. Looking through the lens of resilience as a dynamic process, parasympathetic dominance in resilient individuals may be the result of a biological set point change by anticipatory oscillation within a changing environment as recently suggested [56,57].

This study has several strengths, including its high level of standardization and the repeated administration of four standardized psychosocial stress inductions. Although repeated exposure to the same stress paradigm could potentially drive habituation effects through task familiarity, this risk was minimized by varying job descriptions, panel members, and arithmetic tasks across [34]. Thus, the observed habituation is unlikely to be solely attributable to task repetition; however, to establish this with greater certainty, the role of task familiarity should be more explicitly addressed in future studies (see next paragraph). These rigorous procedures enhance the reliability and validity of the study's findings. By investigating the influence of resilience on the habituation of indicators of the ANS using a highly standardized psychosocial stressor applied four times, a high degree of objectivity was achieved. The resulting findings add additional physiological results to the body of research on the influence of resilience on habituation besides the already found influence of resilience on the habituation of the HPA axis, cardiovascular recovery and electrodermal response [14,15,17].

However, there are limitations to consider. One limitation is the cross-sectional measurement of resilience using the Brief Resilience Scale (BRS), which assesses resilience as a trait rather than a state [58]. Future studies to test the influence of resilience on habituation rather should apply the modern dynamic resilience concept described earlier in the theory [43]. The advantage is the possibility of making influences on resilience directly testable, as it is assumed that resilience changes dynamically. Resilience is measured longitudinally at best, so as not to miss a significant stressor in retrospect [59]. Furthermore, the modern concept makes it easier to examine the influence of physiological mechanisms on resilience and thus identify possible resilience mechanisms to prevent mental illness [5]. Habituation could be such a possible mechanism present in resilient individuals and should therefore be tested on the modern resilience concept. A key limitation of the present study is the absence of respiration measures. As emphasized in recent reporting standards [60,61], respiration strongly influences HRV, particularly frequency-domain indices, and can act as a confounding factor in interpreting autonomic regulation. Without respiratory data, it is difficult to determine the extent to which observed HRV changes reflect true parasympathetic modulation versus respiration-driven variability. Future research should therefore incorporate respiration monitoring alongside HR and HRV to allow for more precise interpretation of autonomic dynamics under stress. Furthermore, participants performed the speech and mental arithmetic tasks (section 3, 4) of the TSST in a standing position, whereas section 1, 2, 5 and 6 were conducted in a seated position; therefore, body posture-related changes in heart rate and heart rate variability may have contributed to the observed physiological responses [60]. Future studies should also consider using shorter time intervals than 5 min, known as ultra-short sequences, as this approach has provided fine-grained analyses of stress in recent studies [62,63]. Another limitation of the present study is that different subsamples were used to assess distinct psychophysiological stress systems, which prevented the joint analysis of multiple markers within the same individuals. As a result, interactions between endocrine, autonomic, and other physiological responses could not be examined. Future studies should aim to assess multiple systems concurrently within the same sample, for example by combining cortisol, heart rate variability, and immunological markers, in order to provide a more integrated understanding of the stress response. Future studies should examine how different emotion regulation strategies moderates the effect of resilience on both acute and recurrent stress responses. A further limitation is that the present study included only young healthy males, which limits the generalizability of our findings. Future research should include women and control for menstrual cycle phase to examine whether the observed associations between resilience, habituation, and autonomic reactivity are consistent across sexes. Another limitation is the absence of a control condition, which makes it difficult to fully separate stress-specific habituation from unspecific influences such as task familiarity or participant expectations. This is a common limitation of traditional laboratory studies proposed by Hughes et al. [16]. Future studies could address this by incorporating control groups (e.g., repeated exposure to non-stressful but similar tasks), by changing the physical location across measurement days, or by varying stressor novelty to test whether habituation generalizes across different stress paradigms.

In summary, this study adds important evidence to the literature by examining the influence of resilience on indicators of autonomic nervous system (ANS) habituation over repeated stress exposures using a standardized psychosocial stressor. While previous research has linked resilience to ANS responses after a single stress event, our findings demonstrate that resilience may plays a role in repeated stress adaptation, seen in habituated heart rate responses and reduced physiological activation over time. By exploring resilience's effects on autonomic recovery and habituation, the study suggests that resilience may serve as a protective factor, contributing to adaptive regulation and potentially lowering allostatic load over repeated stress events.

## CRediT authorship contribution statement

**Christoph Rösner:** Writing – original draft, Project administration, Formal analysis. **Husein Maryam:** Data curation. **Oliver Tüscher:** Supervision, Conceptualization. **Katja Petrowski:** Writing – review & editing, Writing – original draft, Supervision, Conceptualization.

## Funding

This project has received funding from the Leibniz Institute for Resilience Research. Oliver Tüscher received funding from the European Union’s Horizon 2020 research and innovation programme under grant agreement No 777084 (DynaMORE project), from the Deutsche Forschungsgemeinschaft (DFG grant CRC 1193, subproject C04) and from the State of Rhineland-Palatinate (Stiftung Rheinland-Pfalz für Innovation, DRZ programme).

## Declaration of competing interest

All authors have nothing to declare.
