# Supplementary material for: Influence of resilience on autonomic nervous system habituation to repeated stress exposure: Insights from heart rate variability and heart rate response
Source: Compr Psychoneuroendocrinol. 2026 Apr 25;26:100349. doi: 10.1016/j.cpnec.2026.100349 (PMC13138195; doi:10.1016/j.cpnec.2026.100349)
Supplement: Multimedia component 1 [file mmc1.docx]

**Supplementary Material: Detailed distributions of sports activity and sleep variables**

| **Sports per week (hours)** | **Count** | **% of total** | **Cumulative %** |
| --- | --- | --- | --- |
| 0 | 1 | 2.0% | 2.0% |
| 1 | 6 | 12.0% | 14.0% |
| 2 | 5 | 10.0% | 24.0% |
| 3 | 6 | 12.0% | 36.0% |
| 4 | 9 | 18.0% | 54.0% |
| 5 | 9 | 18.0% | 72.0% |
| 6 | 7 | 14.0% | 86.0% |
| 7 | 1 | 2.0% | 88.0% |
| 8 | 1 | 2.0% | 90.0% |
| 9 | 1 | 2.0% | 92.0% |
| 10 | 1 | 2.0% | 94.0% |
| 12 | 2 | 4.0% | 98.0% |
| 21 | 1 | 2.0% | 100.0% |

| **Type of sleep problems** | **Count** | **% of total** | **Cumulative %** |
| --- | --- | --- | --- |
| both (sleep onset and maintenance problems) | 2 | 12.5% | 12.5% |
| sleep maintenance problems | 2 | 12.5% | 25.0% |
| sleep onset difficulties | 2 | 12.5% | 37.5% |
| sleep apnea | 1 | 6.3% | 43.8% |
| sleep onset problems | 2 | 12.5% | 56.3% |
| mild sleep onset problems (without subjective distress) | 1 | 6.3% | 68.8% |
| none | 2 | 12.5% | 81.3% |
| not specified (/) | 1 | 6.3% | 62.5% |
| not specified (-) | 2 | 12.5% | 93.8% |
| none (capitalized entry) | 1 | 6.3% | 100.0% |
